# Combining the HYM (Healthy Young Men’s) Cohort Study and the TRUTH (A Trans Youth of Color Study): Protocol for an Expanded Mixed Methods Study Renewal

**DOI:** 10.2196/39232

**Published:** 2022-11-03

**Authors:** Danny Azucar, Joshua A Rusow, Lindsay Slay, Mariam Taiwo, Aracely Rodriguez, Ali Johnson, Sam Calvetti, Deja Wright, Su Wu, Bethany Bray, Jeremy T Goldbach, Michele D Kipke

**Affiliations:** 1 Division of Research on Children, Youth, and Families Department of Pediatrics Children’s Hospital Los Angeles Los Angeles, CA United States; 2 Institute for Health Research Policy, University of Illinois at Chicago Chicago, IL United States; 3 Brown School of Social Work, Washington University in St. Louis Missouri, MO United States; 4 Department of Population and Public Health Sciences Keck School of Medicine University of Southern California Los Angeles, CA United States

**Keywords:** young men who have sex with men, HIV prevention, intersectionality, gender-nonconforming, multiple stigma

## Abstract

**Background:**

As we enter the fifth decade of the AIDS epidemic, health researchers and AIDS activists reflect both on the progress that has been made and the importance of continued prevention efforts for those most at risk. As HIV infection rates continue to fluctuate across communities, a trend has emerged with new HIV infections becoming increasingly concentrated—with cascading effects—among people aged <30 years, from marginalized racial and ethnic groups, and who are sexual or gender minorities.

**Objective:**

In this paper, we discuss the renewal of the Healthy Young Men’s (HYM) Cohort Study and the addition of a subcohort—TRUTH: A Transgender Youth of Color Study. The overarching aim of our renewed study was to inform new intervention strategies; understand linkage to care; and examine changes over time with respect to minority-related stress and intersectional identities and their relationship with substance use, mental health, and HIV risk. Findings from this study will help to inform the development of new interventions designed to engage African American and Black and Latino young men who have sex with men (YMSM) and transgender and gender minority youth in the HIV prevention and care continua and to reduce risk by addressing pathways of minority-related stress and intersectional stigma.

**Methods:**

Longitudinal study (baseline and follow-up assessments every 6 months for a total of 8 waves of data collection) is ongoing with reconsented cohort from the last iteration of HYM Cohort Study. This study protocol includes self-report survey, collection of urine to assess recent use of illicit drugs, and collection of blood and rectal and throat swabs to test for current sexually transmitted infection and HIV infection. An additional sample of blood and plasma (10 mL for 4 aliquots and 1 pellet) is also collected and stored in the HYM Cohort Study biorepository for future studies. This mixed methods study design includes collection of triangulated analysis of quantitative, qualitative, and biological measures (ie, drug use, sexually transmitted infection and HIV testing, and adherence to antiretroviral therapy among participants who are HIV+) at baseline and every 6 months.

**Results:**

As of February 2022, participants from the past 4 years of the HYM Cohort Study and TRUTH: A Transgender Youth of Color Study Cohort have been reconsented and enrolled into the renewal period of longitudinal data collection, which is projected from summer of 2020 until summer of 2025. Recruitment is ongoing to reach our target enrollment goal of YMSM and transgender minority youth.

**Conclusions:**

The findings from this study are being used to inform the development of new, and adaptation of existing, evidence-based HIV prevention interventions designed to engage populations of transgender and gender minority youth and YMSM in the HIV prevention and care continua.

**International Registered Report Identifier (IRRID):**

DERR1-10.2196/39232

## Introduction

### Disparities With HIV in the United States

The US Department of Health and Human Services has proposed an end to the HIV epidemic in the United States by 2030; this will require flexible, yet targeted, strategies that successfully segment people based on age, demographic characteristics, and location. As HIV infection rates continue to fluctuate across communities, a trend has emerged with new HIV infections becoming increasingly concentrated—with cascading effects—among people aged <30 years, from marginalized racial and ethnic groups, and who are sexual and gender minorities [1,2].

Young men who have sex with men (YMSM) account for most new HIV infections each year. Recent data highlight that, in 2018, 21% of new HIV diagnoses were in people aged between 13 and 24 years, of whom 92% reported male-to-male sexual contact as their mode of HIV infection [3]. These numbers are even more alarming among YMSM of color. Of all new HIV cases among African American and Black men, 20% occur in those aged 13 to 24 years, and 39% occur in those aged between 25 and 35 years [4]. Furthermore, although overall infection rates decreased among the general Latino population from 2005 to 2014, new infections among Latino YMSM aged between 13 and 24 years increased by 87% [5]. Although the overall incidence of HIV infection has decreased in the United States, disparities among youth, particularly African American and Black and Latino YMSM, persist [6,7].

### YMSM and HIV Risk Profiles

The burden of HIV among YMSM is rooted in the interaction of social, economic, and demographic inequities that enhance their risk for HIV. YMSM report more difficulty in accessing health care, anticipated mistreatment from health care providers, and frequent experiences with stigma, which may lead to psychosocial challenges such as increased substance use and involvement in high-risk behaviors [6,8,9]. YMSM of color are especially vulnerable to multiple forms of stigma (eg, discrimination, racism, family rejection, and homophobia) that are influenced by their race, ethnicity, or sexual identity and interact to produce unique HIV risk profiles for individuals [10-12]. For example, African American and Black YMSM report high levels of institutionalized and sexualized racism and discrimination, which puts them at increased risk for internalized homophobia, maladaptive coping, and other mental health issues, which are further associated with illicit drug use and sexual risk taking [13-15]. Among Latino YMSM, who experience great demands to meet traditional gender roles, *machismo* is associated with elevated rates of unprotected sex [16,17].

Stigma also affects YMSM’s engagement in and adherence to the HIV prevention continuum—by discouraging them from accessing information, preventing them from getting tested, and hindering their access to breakthrough biomedical interventions [18]. For example, uptake of pre-exposure prophylaxis (PrEP), a daily regimen of 2 oral antiretroviral drugs taken as a single pill that has been proven to be up to 97% effective in reducing the risk of acquiring HIV [19], is lowest among youth despite their demonstrated need [6,20]. Compared with men who have sex with men (MSM) in general, YMSM are less aware of their HIV serostatus, less likely to engage in and adhere to antiretroviral therapies (ARTs), and less likely to use PrEP; uptake is at 9.4% among YMSM and 19.9% for all MSM [21,22].

Furthermore, African American and Latino YMSM are 2 subgroups with the least likelihood of engaging in PrEP [23,24], suggesting that the efficacy of current HIV prevention efforts is reduced in YMSM who are burdened by multiple forms of co-occurring stigma [7,18].

### Transgender Minority Youth and Increased Risk for HIV

Compared with YMSM, transgender and nonbinary youth of color, or transgender and gender minority youth (TGMY), experience high levels of multiple forms of stigma (eg, low income, isolation, safety concerns, and discrimination) that place them at great risk for poor mental health, depression, suicidality (ie, ideation, plan, and attempt), physical and psychological violence, substance use, and HIV acquisition [7,25-29].

TGMY have varied gender identities that differ from their sex assigned at birth [30,31], and although the terminology used to describe TGMY communities is constantly evolving, TGMY generally includes youth who identify with a gender different from the sex assigned to them at birth or youth who express their gender identity in a way that diverges from the traditional male-female gender binary, such as genderqueer, gender-fluid, or nonbinary. TGMY communities also include sexual, racial, and ethnic minority youth.

Data around TGMY’s HIV risk and health profiles remain limited, especially for young transgender men and nonbinary youth. This lack of information is reflected by few existing resources, which hinders TGMY’s ability to negotiate safe sexual interactions with partners and communicate effectively with providers [7,32]. Health care providers also report a lack of preparation to care for TGMY, and many institutions lack policies and routine practices to support the needs of gender minority patients [33,34]. In addition, in some nonprobability community samples of young transgender women, 25% to 43% of transgender women reported experiencing unstable housing or homelessness [35,36], 67% reported engaging in sex work [35], and 31% reported experiencing sexual violence in the past 12 months [37]. TGMY are 3 times more likely than YMSM to experience workplace discrimination [38], with traumatic experiences and suicidality being more prevalent among nonbinary and gender-nonconforming minority youth [27].

Furthermore, transgender adolescents are more likely to have ever had sex and less likely to have used a condom during their previous sexual experience than their cisgender peers [39]. TGMY are more likely to report first sexual intercourse at or before the age of 13 years, intercourse with ≥4 partners, substance use before sexual activity, and unprotected sex during the previous encounter [40]. Among transgender women and men who seroconverted between 2009 and 2014, 36% of transgender women and 23% of transgender men were aged between 13 and 24 years [26]. Taken together, these findings indicate that TGMY are more at risk for HIV infection than cisgender youth, including YMSM [40], and underlines the need for contextual understanding of the multiple forms of stigma faced by TGMY in relation to their HIV risk [32].

### The Healthy Young Men’s Cohort Study and TRUTH—A Transgender Youth of Color Study: Opportunities to Turn the Curve of the HIV Epidemic

Given the need to further understand the unique experiences of African American and Black and Latino YMSM and TGMY, we propose to build on existing studies and longitudinally examine how (1) YMSM and TGMY experience various forms of intersectional identities and stigma over time, as they age; (2) stigma and intersectionality drive risk for substance use, sexually transmitted infection (STI), HIV infection, and mental health and psychosocial disorders; and (3) stigma and intersectionality serve as barriers to engagement in the HIV prevention and care continua, with particular attention to PrEP and ART uptake and adherence. By examining these aims, we hope to identify when and for whom to target interventions aimed at specific determinants and mechanisms of health. This paper describes the protocol for the renewal of our Healthy Young Men’s (HYM) Cohort Study to continue our research with the HYM and TRUTH: A Transgender Youth of Color Study (TRUTH) Cohorts.

In 2015, Children’s Hospital Los Angeles was awarded a grant (U01DA036926) from the National Institute on Drug Abuse (NIDA) to recruit and longitudinally track a cohort of African American and Black and Latino YMSM to better understand how to engage this high-risk population in the HIV prevention and care continuum. With this grant, we launched the HYM Cohort Study and, in 2016, enrolled 448 African American and Black and Latino YMSM and collected 8 waves of data through assessments administered every 6 months from 2016 to 2020. In 2018, we received an administrative supplement to recruit and collect 2 waves of data for a new cohort of transgender, nonbinary, and gender-nonconforming youth. From this, we launched the substudy, TRUTH, which recruited 108 participants via social media and community referrals and collected 3 waves of data through assessments administered every 6 months from 2018 to 2020; study assessments for both HYM and TRUTH included survey data collection, STI and HIV testing, and drug screening every 6 months.

In 2020, our renewal application was awarded. For this next funding cycle (2020-2025), our study will focus on HIV risk and transmission for YMSM and TGMY through the lens of intersecting stigmatized identities, including sexual identity, gender identity, race, ethnicity, immigration status, financial hardship, HIV+ status, mental health, psychiatric disorders, and sex work or transactional sex.

### Study Aims

#### Overview

The overarching aims of this study are to inform new intervention strategies; understand linkage to care; and examine changes over time with respect to minority-related stress and intersectional identities and their relationship with substance use, mental health, and HIV risk. Findings from this study will help to inform the development of new interventions designed to engage African American and Latino YMSM and TGMY in the HIV prevention and care continua and to reduce risk by addressing pathways of minority-related stress and intersectional stigma. The specific aims are the following:

#### Aim 1

Examine African American and Black and Latino YMSM’s engagement in the HIV prevention and care continua and their developmental transitions and trajectories in drug use, STI and HIV infection, health, and psychiatric and mental health comorbidities. We will examine how intersectional stigma (ie, experiences of stigma stemming from multiple intersecting identities) both discourages African American and Black and Latino YMSM from engaging in care and influences their developmental arcs of risk, transmission, and health.

#### Aim 2

Examine TGMY’s engagement in the HIV prevention and care continua and identify shared and unique transitions and trajectories regarding TGMY’s use of drugs, STI and HIV infection, health, and psychiatric and mental health comorbidities. Of particular interest is how these processes are informed by experiences of stigma occurring at the intersection of their multiple, marginalized identities.

#### Aim 3

Serve as a local and national resource for collaborations and dissemination. We will actively participate in and contribute to the NIDA initiative, Collaborating Consortium of Cohorts Producing NIDA Opportunities. We will also partner with key stakeholders (eg, community organizations and policy makers) and collaborate with trainees, early-career faculty, and investigators across the translational spectrum.

### Theoretical Model and Conceptual Framework: Theory of Minority Stress and Intersectionality

Results from the first 4 years of data collection suggest a complicated story that is rooted in minority stress, stigma, and intersectionality of identity and stigma [15]. YMSM and TGMY are located at the intersection of multiple identities, each of which has unique forms of stigma; thus, applying concepts of intersectional identity and stigma to observational studies may provide novel insights into facilitators of and barriers to HIV care.

#### Theory of Minority Stress

This theory has emerged as a framework to explain the relationship between prejudice, discrimination, stigma, and adverse health outcomes (eg, substance use and high-risk behaviors) [41]. Minority stress stems from several social and psychological theoretical orientations and can be described as a relationship between minority and dominant values and the conflict with the social environment experienced by minority group members. This theory posits that sexual and gender minority health disparities in care are caused mostly by stressors induced by hostile, homophobic and transphobic culture, which often results in a lifetime of harassment, maltreatment, discrimination, and victimization, which ultimately affects access and use of care [41].

Minority stress is also associated with depression and traumatic experiences, which are each linked to suicidal ideation and attempt, thus supporting the notion that minority stress is directly and indirectly linked to negative health outcomes through multiple mental health symptom pathways [42]. This literature is consistent with our own study findings demonstrating that racism, homophobia, internalized homophobia, and stigma are all important to understanding risk among TGMY and YMSM [15].

#### Theory of Intersectionality

This theory serves as an extension to the Theory of Minority Stress by examining how multiple aspects of marginalized or stigmatized identities (eg, race, class, sexual and gender identity, or HIV status) potentially affect health-related behaviors and outcomes. Intersectionality, or intersectional identity, describes the compounded effects that multiple marginalized identities have on individuals [43,44]. It considers the ecological factors that affect experiences of stigma, discrimination, and marginalization [25]—and in the context of HIV, provides an important framework for understanding the disproportionate risk among YMSM and TGMY. Intersectionality posits that multiple stigmatized identities such as gender, race, ethnicity, sexual orientation, and socioeconomic status, among others, do not exist independently, but intersect to reflect individual experiences and should therefore be considered simultaneously [18,43,45].

An intersectional perspective is vital to holistically understand how living with multiple stigmatized identities affects individual behaviors and population health outcomes [46]. Intersectionality is an emerging approach to stigma studies that can be used to better understand the experiences of vulnerable groups with multiple stigmatized identities while providing guidance on intervention strategies that can reduce stigma, increase resilience, and improve health [47]. Furthermore, it creates a framework for examining the juncture of multiple stigmatized identities that fall within or across several categories: (1) ≥1 coexisting health conditions such as HIV, drug use, or mental health disorders; (2) demographic sociostructural characteristics, such as race, ethnicity, sexual orientation, gender identity, homelessness, and immigration status; and (3) behaviors and experiences such as drug use and sex work.

## Methods

### Ethics Approval

This study has been reviewed and approved by the institutional review board (IRB) at Children’s Hospital Los Angeles (CHLA-14-00279). Participants were initially recruited from the previous iterations of HYM and TRUTH studies [48]; they were screened for eligibility to continue in the study, and if eligible, they were invited to participate in the study as further described in the following sections. All participants provided written informed consent during a web-based reconsenting visit and were informed that continuing participation in the study is voluntary and that they could exit at any time.

### Study Design

We will use a longitudinal design with data collection occurring every 6 months to examine changes over time with respect to intersectional identities and relationship with HIV and STIs, drug use, and mental health risk. Data collection will include a quantitative survey; qualitative interviews; and collection of biological specimens for rapid HIV tests and STI tests (ie, rectal and throat swabs), urine for recent substance use, blood for syphilis testing, and blood samples for later analysis. HYM and TRUTH Cohort Study will continue to conduct triangulated analysis of quantitative, qualitative, and biological measures (ie, drug use, HIV and STI testing, and adherence to ART for participants who are HIV+) at baseline and every 6 months for a total of 8 waves of data collection scheduled to occur from fall of 2021 to summer of 2025. Furthermore, qualitative substudies will be integrated into the study using the time line follow-back approach on an as-needed basis to further contextualize our quantitative findings.

### Study Participants

In 2016, we recruited a cohort of 448 (n=397, 88.6% HIV– and n=51, 11.4% HIV+) YMSM into the HYM Cohort Study; the sample comprised African American and Black and Latino YMSM who are HIV+ or at high risk for HIV acquisition; 58.9% (264/448) self-identified as Latino, 20.9% (94/448) as African American, and 20.1% (90/448) as multiracial. Mean age at recruitment was 22.3 (SD 2.02) years. We will continue data collection with this existing cohort. To do this, we contacted participants, informed them of the study renewal, and invited them to reconsent via an IRB-approved web-based video conferencing.

Similarly, data collection will continue with the current TRUTH cohort (N=105) using the approach described previously. In addition, we will recruit 250 new TGMY (eg, approximately n=100, 40% transgender women; n=100, 40% transgender men; and n=50, 20% gender-nonconforming and nonbinary youth) to allow for additional data collection and analysis with this understudied community. We are particularly interested in recruiting participants who are substance users (especially methamphetamines and opioids) or living with HIV. Eligibility criteria for additional study recruitment of the HYM and TRUTH Study Cohorts are as follows: (1) aged between 20 and 26 years; (2) self-identify as Black or African American, Latino or Latinx, or a mixed-race individual; (3) self-identify as a cisgender man, transgender individual, or nonbinary individual; (4) lived in the Los Angeles metropolitan area; and (5) have had sex with a penis in the past 12 months.

YMSM and TGMY will be excluded from the study if they (1) do not meet the inclusion criteria, (2) appear to be under the influence of drugs or alcohol during their study visit (research staff is trained to identify objective signs of intoxication), (3) are not able to commit to the study through completion, or (4) are not fluent in English or Spanish as determined during screening and informed consent process.

### Recruitment

Recruitment resumed in October 2021. We continue to recruit participants via referrals from community partners and through social media platforms such as Facebook, Instagram, and Grindr [48].

### Tracking and Retention

This study will continue using the tracking and retention procedures previously used, including incentivized monthly check-ins with participants to identify changes in contact information [48]. Participants receive US $5 per month for reaching out to study staff and alerting them to any changes in contact information. Periodic newsletters are sent to participants to remind them about study-related events, and social events maintain participant engagement throughout the study.

### Community Advisory Board and Youth Community Advisory Board

Community Advisory Boards (CABs) continue to be critical partners in our study conducted with YMSM and TGMY. We will continue to convene a CAB and Youth CAB (YCAB) to inform all aspects of the HYM and TRUTH Cohort Study. CAB and YCAB members include policy makers, HIV prevention and care service providers, advocates, and members of our target population (eg, racially and ethnically diverse YMSM and TGMY). Our CAB and YCAB have been invaluable in helping us to develop or refine our data collection instruments, methods, sample selection and retention efforts; identify resources for participants; interpret study findings; and disseminate findings to local organizations and policy makers. Going forward, we have adapted our CAB and YCAB to include great representation for our study population of YMSM and TGMY; we have confirmed a total of 18 CAB members, who meet quarterly.

### Assessment Measures

#### Overview

We use a core set of measures for the HYM and TRUTH assessments. Surveys require approximately 90 minutes to complete and are self-administered using a secure web-based platform to protect participants’ privacy and reduce response bias, particularly for sensitive questions about sexual behaviors and substance use. Participants who wish to take the survey remotely meet with one of our research associates (RAs) via secure videoconference. During remote survey sessions, participants are allowed to mute their microphone and camera, whereas the RA remains on standby for any participant questions. Participants who come to the office for in-person surveys are brought to a private conference room to ensure privacy and are informed that their RA is on standby to provide immediate support and clarification of questions.

In our latest survey, the separate HYM and TRUTH surveys were combined to yield a single survey with a set of core items that would be presented to all participants. Skip logic was programmed such that specific questions (eg, forms of health care and experiences of discrimination) are presented only to relevant participants. For example, questions regarding experiences with HIV-related care are only asked to participants who have been diagnosed and are living with HIV. Experiences of transgender-related access to health care, discrimination and coping are only presented to participants who identify as transgender or gender expansive (ie, TRUTH Study Cohort participants), whereas questions assessing discrimination and coping pertaining to gay or bisexual men or MSM will only be administered to participants enrolled in HYM Cohort Study. For the 2020 to 2025 cohort, we have retained core measures from the original survey and have included new measures described in the following sections. The HYM+TRUTH survey was significantly modified to reflect the new aims of the renewed study.

#### Discrimination, Stigma, and Identity

Experiences of discrimination and stigma are assessed generally and in relation to either or both race or ethnicity and sexual or gender identity. General discrimination is assessed using the *Everyday Discrimination Scale,* which asks participants about different experiences and whether they attribute those experiences to aspects of their identity including ancestry or national origins, gender, race, age, religion, height, weight, appearance, sexual orientation, education, or income level [49]. The *LGBT People of Color Microaggressions Scale* assesses multiple minority stress [10]. *The Intersectional Discrimination Index* measures anticipated, day-to-day, and directly enacted stigmatizing experiences attributed to how participants describe themselves and how others may describe them [50]. The *Conflicts in Allegiances Scale* assesses how cultural identity, race, or ethnicity interacts with sexual or gender identity [51]. Experiences of homophobia and racism are assessed using subscales of the *Homophobia, Racism and Poverty Scale* [52]. HIV-related stigma is measured using a revised and brief measure of stigma toward youth who are HIV+ [53]. Anticipated stigma is assessed using the *Heightened Vigilance Scale* [54]. In addition, we measure multiple internalized stigmas including internalized racism [55], transphobia [56], and homonegativity [57,58]. Finally, we measure positive identity including community connectedness [59]; positive racial and ethnic identity [60]; positive lesbian, gay, bisexual, and transgender identity [61]; and gender expression [62].

#### Behavioral and Mental Health

We measure several constructs that are possible mediators or moderators—and known correlates—of sexual health and HIV. Impulsivity is captured using the *Barratt Impulsiveness Scale Short-Form* [63]. We assess emotion regulation [64] and partner violence [65]. Coping is assessed using the *Brief COPE* [66], subscales of the *Racism-Related Coping Scale* [67], and coping items related to sexual and gender identity [68]. Finally, social support is measured using both the original *Multidimensional Scale of Perceived Social Support* and an adapted version that enquires about lesbian, gay, bisexual, transgender, and queer and racial and ethnic community support [69]. Furthermore, TRUTH-specific assessments include measures of use of hormones and silicone and items regarding support from family and peers for TGMY identity.

#### Biorepository

We will continue to maintain a biorepository with blood samples and rectal swabs, which are collected annually from our participants who are HIV– and every 6 months from participants who are HIV+. Specimen collection includes 10 mL EDTA anticoagulated whole blood samples and 2 rectal swabs. Blood specimens are processed to harvest plasma and a cellular pellet. Plasma is then divided into 4 separate aliquots and stored frozen at −80 °C. A red blood cell, buffy coat pellet is harvested and stored for future nucleic acid extraction. This cellular material will be made available to investigators for future studies. All specimens are stored in a secure, password-protected database; their position in storage is noted (eg, rack, box, and position), and they are deidentified from all study participant information. Regarding substance use, we will collect urine samples at each visit to test for the metabolites of methamphetamines, cocaine, ecstasy, marijuana, and opiates using the Integrated E-Z Split Key Cup II-5 Panel (Innovation Laboratories), which can detect drugs from 1 to 4 days after use, except for chronic marijuana use, which can be detected for up to 30 days.

#### STI Status

Participants will self-collect rectal and pharyngeal specimens for *Neisseria gonorrhea* and *Chlamydia trachomatis* nucleic acid amplification testing (Hologic) at a Clinical Laboratory Improvement Amendments–compliant clinical laboratory. This will occur in our field offices or at a partnering test site. Participants who test positive will be referred to a HIV and STI clinic and treated (along with their recent partners) according to the Center for Disease Control and Prevention and Los Angeles County guidelines. Syphilis testing will be performed using whole blood collected via venipuncture (or fingerstick) using rapid plasma regain and treponemal antibody testing. Specimens will be coded with participants’ unique code and securely sent to the Public Health Laboratories for testing and processing. Those with syphilis infection will be referred and treated at one of our partner clinical sites according to the Center for Disease Control and Prevention and Los Angeles County guidelines. STI status will be assessed at each of the 6-month assessments.

#### HIV Status

HIV status will be assessed at each of the 6-month assessments, and it will be done using the Integrase Strand Transfer Inhibitors HIV-1 and HIV-2 antibody test (Biolytical Laboratories). This will be completed by field staff who have been trained in HIV testing procedures and counseling. We have integrated HIV testing and linkage to care for those who test positive, given the importance of ensuring that youth who are HIV+ are linked with care. Participants who test positive for HIV will be offered information about local resources and referred to clinical care sites for confirmatory testing, further diagnostic evaluation, and care.

#### Studies on Stress-Related Biomarkers

We have been conducting studies to understand the relationship between stress and stressful life events, drug use, and HIV risk. Before February 2022, we have administered the Strain and Adversity Inventory (STRAIN) to 66.9% (300/448) of our cohort participants. STRAIN is an instrument recommended by the National Institute of Mental Health Research Domain Criteria that efficiently and reliably assesses a person’s cumulative exposure to stress over the life course. We hypothesize that stress, including racism and discrimination, will be significant predictors of drug use and HIV risk in our cohort. We will continue these analyses and examine the relationship between STRAIN scores and stress-related biomarkers, including biomarkers of inflammation, genome-wide transcriptional profiling, and biological aging.

#### Time Line Follow-Back Method Assessment

We will use an adapted time line follow-back method assessment to better understand YMSM’s and TGMY’s experiences in the HIV care continuum. This method uses memory aids such as calendars and *anchor days* to assist respondents in creating a daily diary for specific behaviors or events [70]. This technique has been found to have acceptable reliability and validity when measuring constructs such as substance use and sexual behavior and similar coefficients to data obtained from more conventional assessments such as single-item survey questions. This technique will be used to complete substudies that will be used to contextualize specific experiences including (1) heavy substance use (to understand social cues or other triggers for this use), (2) inconsistent use of care and challenges in accessing health services (to address structural barriers to care), (3) HIV testing (to identify why YMSM and TGMY are not testing as recommended), (4) dropping out of care, and (5) challenges in adhering to ART medications and achieving viral suppression.

#### Pandemic Impact Scale

In addition, a subset of our existing cohort was administered the Pandemic Impact Scale [71], which provides a holistic assessment of the impact of the pandemic on participants’ lives, including work and employment, education and training, home life, social activities, economic stability, emotional health and well-being, physical health problems, physical distance and quarantine, infection history, and positive changes owing to the pandemic. Findings from the Pandemic Impact Scale will be reported in a separate paper outlining the impact that the COVID-19 pandemic has had on our overall study participants.

### Data Analysis

Univariate statistics will be used to examine and chart the characteristics of the cohorts and subgroups within them (eg, race and ethnicity subgroups and gender identity subgroups), including demographics, STI and HIV incidence by pathogen and anatomical site, substance use from self-report and urine samples, use of HIV testing and prevention services, health care use and engagement, use of biomedical HIV prevention (eg, PrEP) for those who are HIV–, and adherence to ART for those who are HIV+. Bivariate analysis will be used to examine relations among variables of interest. For constructs assessed using multiple items, factor analysis will be used to confirm the scales, and summary scale scores will be created. Psychometric analysis of the developed measures will be performed; exploratory analyses will be used immediately after alteration or creation to explore redundancy or inadequacy of items; and confirmatory analysis will be used at later waves to examine validity, reliability, and stability of the composite scores.

In addition, Latent Class Analysis provides a parsimonious way to identify specific behaviors characterized by particular patterns of responses and to link subgroup membership to predictors and outcomes. We will use Latent Class Analysis to examine the following: (1) patterns of co-occurring substance use, HIV, STI, and mental health comorbidities; characteristics of engagement in care; and how pattern membership changes over time; (2) patterns of multiple intersecting identities and changes over time; (3) patterns of multidimensional interconnected experiences of stigma and changes over time; (4) how associations among HIV and STIs, substance use, mental health, and engagement in care are informed by intersectionality; and (5) differences between African American and Black and Latino YMSM and TGMY in complex intersectionality processes.

### Data Management

All data and biological samples collected for this study will remain confidential; all participants’ personal information will be coded using a combination of numbers and letters such that the data collected cannot be linked back to the study participants. All personal identifiable information will be removed from self-reported assessments and biological specimens, and any findings in future scientific journals or conferences will be reported in aggregate form and only after the data have been deidentified. All participant data will be deidentified from participant information. All self-reported and collected data will remain in a password-protected database, and all biological specimens will be entered into a secure, password-protected database and be accessible only to the study staff.

## Results

Funding for the renewal project began in June 2020, and IRB approval to increase enrollment was obtained on July 9, 2021. As of February 2022, participants from the past 4 years of the HYM Cohort Study and TRUTH Study Cohort have been reconsented and enrolled into the renewal period of longitudinal data collection, which is projected from summer of 2020 to summer of 2025. Recruitment is ongoing to reach our target enrollment goal of 700 YMSM and TGMY. As of September 30, 2022, in total, 68.4% (479/700) participants have been enrolled or reconsented into the renewal project. Assessments will be conducted every 6 months until the end of this project period, in July 2025.

## Discussion

### Principal Findings

This paper describes the renewal of a longitudinal study with efforts to combine 2 different cohorts of YMSM and TGMY into a unified cohort, with distinct study measures adapted for each. We discuss the renewal period and the adoption of new measures to assess psychosocial characteristics that align with our current study aim—adopting a framework of intersectionality to develop effective interventions for YMSM and TGMY, who may experience multiple forms of stigma owing to their multiple minority identities.

As we broach the fifth decade of the AIDS epidemic in the United States, health researchers and AIDS activists reflect on both the progress that has been made in reducing the incidence of AIDS and the importance of continued prevention efforts. Although significant advances have been made in reducing the incidence of new infections, recent studies have demonstrated that some groups continue to have elevated exposure to HIV transmission risk. YMSM and TGMY are such groups, and more traditional risk reduction efforts do not appear to be effectively reaching this population. One of the reasons why more traditional HIV prevention efforts have failed among these young people is that such efforts often fail to incorporate broad familial, social, cultural, and community factors that influence their lives. Very few proven interventions are available that have been targeted for these groups. The importance of this study rests on its potential to generate appropriate and meaningful HIV prevention efforts targeting YMSM of color and TGMY and to integrate current understanding of a range of psychosocial factors and their influence on HIV risk and protective behaviors, particularly drug use and sexual practices.

### Conclusions

We will work to ensure that our cohort serves as a national resource for collaborations and dissemination (aim 3). Our cohorts provide unique opportunities for researchers and interventionists to assess and test how we can reach our national goals with 2 key populations: African American and Black and Latino YMSM and TGMY. In particular, the proposed mixed methods study may offer important insights into the broad array of interpersonal, social, and cultural relationships in young people’s lives that underlie their involvement in drug use and HIV risk and protective behavior. This study will provide important information that can be used to develop gender-specific and age-appropriate substance use and HIV prevention and harm reduction efforts.

## Abbreviations

ART: antiretroviral therapy
CAB: Community Advisory Board
HYM: healthy young men
IRB: institutional review board
MSM: men who have sex with men
NIDA: National Institute on Drug Abuse
PrEP: pre-exposure prophylaxis
RA: research associate
STI: sexually transmitted infection
STRAIN: Strain and Adversity Inventory
TGMY: transgender and gender minority youth
TRUTH: TRUTH: A Transgender Youth of Color Study
YCAB: Youth Community Advisory Board
YMSM: young men who have sex with men

## Data Availability

The data sets generated and analyzed during this study are available from the corresponding author upon reasonable request.
